# Transcription factors define the neuroanatomical organization of the medullary reticular formation

**DOI:** 10.3389/fnana.2013.00007

**Published:** 2013-05-14

**Authors:** Paul A. Gray

**Affiliations:** Department of Anatomy and Neurobiology, Washington University School of MedicineSt. Louis, MO, USA

**Keywords:** brainstem, transcription factors, reticular formation, fate-mapping, development, rhombomeres, hindbrain

## Abstract

The medullary reticular formation contains large populations of inadequately described, excitatory interneurons that have been implicated in multiple homeostatic behaviors including breathing, viserosensory processing, vascular tone, and pain. Many hindbrain nuclei show a highly stereotyped pattern of localization across vertebrates suggesting a strong underlying genetic organization. Whether this is true for neurons within the reticular regions of hindbrain is unknown. Hindbrain neurons are derived from distinct developmental progenitor domains each of which expresses distinct patterns of transcription factors (TFs). These neuronal populations have distinct characteristics such as transmitter identity, migration, and connectivity suggesting developmentally expressed TFs might identify unique subpopulations of neurons within the reticular formation. A fate-mapping strategy using perinatal expression of reporter genes within *Atoh1*, *Dbx1*, *Lmx1b*, and *Ptf1a* transgenic mice coupled with immunohistochemistry (IHC) and *in situ* hybridization (ISH) were used to address the developmental organization of a large subset of reticular formation glutamatergic neurons. All hindbrain lineages have relatively large populations that extend the entire length of the hindbrain. Importantly, the location of neurons within each lineage was highly constrained. *Lmx1b*- and *Dbx1*- derived populations were both present in partially overlapping stripes within the reticular formation extending from dorsal to ventral brain. Within each lineage, distinct patterns of gene expression and organization were localized to specific hindbrain regions. Rostro-caudally sub-populations differ sequentially corresponding to proposed pseudo-rhombomereic boundaries. Dorsal-ventrally, sub-populations correspond to specific migratory positions. Together these data suggests the reticular formation is organized by a highly stereotyped developmental logic.

## Introduction

The organization of the hindbrain has been of significant interest for well over 100 years. Johnson proposed it could be functionally divided dorso-ventrally into four discrete regions based on the location of afferent and efferent projections, and that this organization was a shared characteristic of vertebrate lineages (Johnson, 1902). Based on comparative analysis in embryonic vertebrates, this organization was extended by Bergquist and Källěn (1954) and others [see review by Nieuwenhuys (2011)] to divide the neuroaxis rostrocaudally into specific neuromeric regions. The volumes circumscribed by these rostro-caudal and dorso-ventral divisions may represent functional “morphological units” that are the consequence of neural migration within a unit (Nieuwenhuys, 2011).

Recent work describing the molecular development of the hindbrain found that the rostro-caudal boundaries of a subset of these regions, now termed rhombomeres, correlate closely with the expression during early development of highly evolutionarily conserved transcription factors (TFs) involved in patterning of the body plan, homeobox (Hox) genes (Krumlauf et al., 1993). Rhombomeric boundaries, however, have only been clearly described for pontine hindbrain regions (R1–R6) (Manzanares et al., 1999; Voiculescu et al., 2001). The caudal R6 boundary and the boundaries of more caudal rhombomeres within the mouse hindbrain are unclear. Work using chick-quail chimeras suggests the existence of at least four “pseudo-rhombomeres” (pr7–10) within the medulla based on the migration and formation of multiple medullary nuclei (Cambronero and Puelles, 2000; Marin et al., 2008). Currently, no genes have been identified in the early developing mouse embryo whose expression correlates with these boundaries. Similarly, based on patterns of TF expression, the hindbrain can be divided dorso-ventrally into at least 16 domains, consistent, at a first approximation, with the four divisions proposed by Johnson (Johnson, 1902; Sieber et al., 2007; Gray, 2008; Storm et al., 2009).

The extension of these organizational heuristics into the postnatal or adult hindbrain has several major limitations. First, the initial hypotheses were based on the analysis of highly identifiable populations that only represent a subset of hindbrain neurons, such as cranial motor pools or the target zones of sensory input (Altman and Bayer, 1980a,b; Kalia and Fuxe, 1985; Vanderhorst and Ulfhake, 2006). Second, they assume that neurons largely remain within their morphological unit and their formation is strictly a function of cellular migration along a radial glial scaffold. Exceptions, such as the precerebellar pontine nucleus and inferior olive, were found to be derived from discrete developmental domains at or near the dorsal surface, the rhombic lip, which then underwent radial migration to positions on the ventral surface (Dymecki and Tomasiewicz, 1998; Nichols and Bruce, 2006; Yamada et al., 2007; Rose et al., 2009b; Storm et al., 2009).

The extent to which other populations of hindbrain neurons, especially within the medulla, share a similar organizational logic has only recently started to be addressed. For example, our understanding of aminergic populations has greatly expanded due to recent analysis of the TFs necessary for their formation (Qian et al., 2001; Cheng et al., 2003; Ding et al., 2003; Zhao et al., 2006). These populations are derived from very specific rostro-caudal and dorso-ventral developmental domains. One major difference was that unlike previously studied populations, several catecholaminergic populations undergo significant migration (Kalia et al., 1985a,b,c). To add to this complexity, a significant number of hindbrain neurons make up the “reticular formation,” i.e., they do not lie within clearly identified nuclei, defined either by neuronal density or gene expression. Brodal, in his seminal description of the reticular formation, defined it as “those areas of the brain stem which are characterized structurally by being made up of diffuse aggregations of cells of different types and sizes, separated by a wealth of fibers travelling in all directions” (Brodal, 1957). Thus, whether the hindbrain has an underlying genetic logic that applies to all neurons is unknown.

A number of large-scale gene expression projects have coupled advances in genomics with anatomy providing a new wealth of information (Gray et al., 2004; Lein et al., 2007; Diez-Roux et al., 2011). This information, however, is limited by our inability to uniquely identify the majority of neuronal populations in the brain. TFs are proteins that directly bind DNA to control the expression of large numbers of genes. While many TFs are either ubiquitious or controlled by activity dependent mechanisms, some are essential for the specification and maintenance of cellular identity (Gray et al., 2004; Gray, 2008). In fact, one very influential idea hypothesizes that the particular combination of TFs expressed during development is the defining characteristic that specifies neural populations (Briscoe et al., 2000; Baumgardt et al., 2007). Because of this, TFs have been extensively used as markers for the identification of specific neural populations. TFs, like all genes, are expressed in multiple cellular populations, but because of their unique function, they provide a unique context for understanding gene expression.

The mouse genome contains ~1500 putative TFs all of which have their own temporal and spatial pattern of expression within the brain (Gray et al., 2004). Of these, only a subset of TFs has been shown to be both spatially limited to and essential for the development of specific subpopulations of neurons. One consequence of the limited number of lineage specific TFs within the mouse genome, compared to the large number of discrete neuronal lineages, is that nearly all TFs are expressed in multiple distinct populations. These populations, however, are often far enough apart within the brain for relatively easy discrimination. In addition, each lineage has unique patterns of TF co-expression. Thus, individually TFs label diverse populations, but within limited regions and/or in combination with other TFs, they define small populations of neurons. These “lineage-specific” TFs are highly useful for the identification of discrete subpopulations of neurons from development into adulthood (Vandunk et al., 2011; Miller et al., 2012). Many TFs, however, are expressed either only transiently or lack high quality commercially available antibodies limiting their use for extensive analysis. One method to overcome these limitations has been to mark specific subsets of neurons by using transgenic mice expressing reporter proteins that either directly, or via Cre-mediated recombinase activity, label cells for extended periods and allow for ease of visualization (Srinivas et al., 2001; Dymecki et al., 2002).

The medullary reticular formation contains a large number of excitatory and inhibitory neurons involved in the control of breathing (Feldman et al., 2013), vasomotor tone (Dampney et al., 1982; Guyenet, 2006; Abbott et al., 2012), pain (Esser et al., 1998; Almeida et al., 2002; Lima and Almeida, 2002), somatomotor functions (Brodal, 1952; Strack et al., 1989; Jansen et al., 1995; Geerling and Loewy, 2008), and a variety of other homeostatic processes. It is also known to have extensive local and distal connectivity. Work from several groups has identified developmental genes important for multiple aspects of these important systems. For example, the TFs *Lmx1b* (lim homeobox transcription factor 1b) and *Phox2b* (paired mesoderm homeobox 2b) are fundamental for the formation of multiple brainstem populations including glutamatergic neurons of the nucleus of the solitary tract (NTS) and brainstem catecholaminergic neurons (Yang et al., 1998; Pattyn et al., 1999, 2000a,b; Brunet and Pattyn, 2002; Cheng et al., 2003; Ding et al., 2003; Dubreuil et al., 2009; Ramanantsoa et al., 2011). *Phox2b* is a homeobox TF also involved in the specification of visceral sensory input and motor output (Brunet and Pattyn, 2002; Dauger et al., 2003). *Lmx1b* is a homeobox TF also involved in specification of sensory populations as well as serotonergic neurons (Cheng et al., 2003; Ding et al., 2003, 2004; Zhao et al., 2006). Both *Phox2b* and *Lmx1b* expression are defining characteristics of several hindbrain lineages. *Dbx1* (developing brain homeobox 1) is essential for the formation of glutamatergic preBötzinger Complex (preBötC) respiratory neurons as well subsets of neurons from the spinal cord to the cortex (Pierani et al., 2001; Bouvier et al., 2010; Gray et al., 2010). *Ptf1a* (pancreas specific transcription factor 1a) is a TF important for the specification of a large percentage of hindbrain and spinal cord inhibitory neurons as well as inferior olive neurons (IO) (Glasgow et al., 2005; Hoshino et al., 2005; Yamada et al., 2007; Brohl et al., 2008). Combined with *Lbx1* (ladybird homeobox 1), *Ptf1a* is vital for the formation of Bötzinger Complex (BötC) glycinergic respiratory neurons (Pagliardini et al., 2008). Similarly, *FoxP2* (forkhead box P2) is a leucine zipper transcription factor implicated in motor control in humans (Lai et al., 2001). Previous analyses found *FoxP2* is expressed in small subsets of spinal cord, medullary, and pontine neurons in lineage specific manner (Gray, 2008; Prasad et al., 2008; Geerling et al., 2011; Miller et al., 2012). *Dbx1* is a homeobox TF necessary for the formation of the preBötC (Pierani et al., 2001; Bouvier et al., 2010; Gray et al., 2010). Neurons derived from lineages expressing these various TFs, however, are broadly localized throughout the hindbrain.

**Figure 1:**
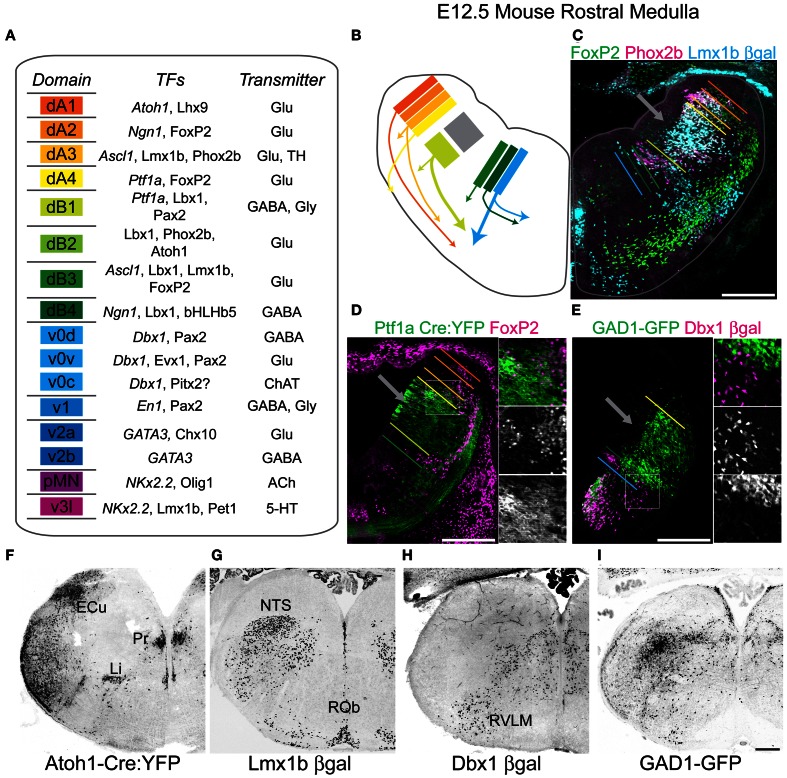
**Genetic diversity of hindbrain neurons. (A)** Schematized diagram describing brainstem progenitor domains for eight dorsal (dA1–dB4) and 8 ventral progenitor populations (v0–v3l) based on their relative dorso-ventral location during neurogenesis (left). Partial list of transcription factors expressed within progenitors (italics) or post-mitotic neurons within each domain (middle). Neurotransmitter identity of neurons derived from each domain (right). **(B)** Cartoon showing partial migratory path of ventral medulla neurons in embryonic mouse brainstem. Colors correspond to domains in **(A)**. Thick arrows correspond to populations migrating to ventrolateral medulla. **(C)** Pseudo-color confocal image of FoxP2 (green), Pho×2b (magenta), and βgal (cyan) expression in an E13.5 *Lmx1b*-βgal mouse showing the origin and migration of hindbrain neurons. Colored bars correspond to boundaries of progenitor domains from **(A)**. **(D)** Pseudo-color confocal image of FoxP2 (magenta) and YFP (green) expression in E13.5 *Ptf1a*-Cre; R26 YFP medulla. Region in box is expanded to right showing overlapping (top), FoxP2 (middle), and YFP (bottom) expression. Colored bars correspond to boundaries of progenitor domains from **(A)**. **(E)** Pseudo-color confocal image of intrinsic GFP (green) and βgal (magenta) expression from E13.5 GAD1-GFP; *Dbx1*-βgal double transgenic mouse. Region in box is expanded to right showing overlapping (top), βgal (middle), and GFP (bottom) expression. Inverted confocal mosaic image showing localization of lineage derived neurons in hemisections from rostral medulla from P0 *Atoh1* **(F)**, *Lmx1b* **(G)**, *Dbx1* **(H)**, and *GAD1* **(I)** transgenic mice. Scale bar = 500 μm.

During early neurogenesis (~E9–E11 in mouse), the hindbrain neural progenitor zone can be divided into at least 16 dorso-ventral domains based on discrete patterns of TF expression (Figure 1A) (Sieber et al., 2007; Gray, 2008; Storm et al., 2009). Work from a number of laboratories has shown that each hindbrain and spinal cord progenitor domain has the capacity to produce multiple neural subtypes with distinct migratory paths, neurotransmitter phenotypes, and axonal projection patterns (Helms and Johnson, 2003; Glasgow et al., 2005). At present, the final location and function of the vast majority of these neurons are unknown. A number of recent papers in both mice and rats have described the localization of some of the neurons derived from or expressing specific TFs within the hindbrain, including *Atoh1* (atonal homolog 1), *Dbx1*, *Ptf1a*, *Lmx1b*, and *Phox2b* populations (Qian et al., 2001; Lumpkin et al., 2003; Wang et al., 2005; Kang et al., 2007; Yamada et al., 2007; Pagliardini et al., 2008; Rose et al., 2009a,b; Bouvier et al., 2010; Gray et al., 2010). While informative, it has been difficult to integrate these diverse results into a more general understanding of the hindbrain, especially the medulla.

The availability of transgenic mice with genetically encoded reporter genes driven by TF genomic promoter regions allows for the identification of neuron developmental history. I hypothesized that mapping the location of multiple developmental lineages in relation to each other and to medullary glutamatergic neurons present in both the dorsal and ventrolateral medulla may provide insight into the underlying genetic organization of hindbrain interneurons. Using fate-mapping in six transgenic mouse lines that identify the majority of medullary glutamatergic neurons, combined with immunohistochemistry (IHC) and vesicular glutamate transporter *in situ* hybridization (ISH), I provide a map of the locations of specific subpopulations of medullary reticular formation neurons. From these data, I propose the medulla has a clearly understandable anatomical logic based on dorso-ventral developmental origin and rostro-caudal segmentation. Within the reticular formation, excitatory neurons from specific lineages are located in discrete stripe-like domains that only partially overlap. These populations vary along the rostro-caudal axis in a pattern similar to proposed pseudo-rhombomeric boundaries. Moreover, these data suggest that the developmental origins of many reticular formation glutamatergic neurons can be predicted from their location and function.

## Methods

### Mice

We employed *Atoh1*^βgal^/+ (*Atoh1*-βgal) (Ben-Arie et al., 1997), *Atoh1*-Cre (Chen et al., 2002), *Dbx1*^βgal^/+ (*Dbx1*-βgal) (Pierani et al., 2001), Gad1-GFP transgenic (Chattopadhyaya et al., 2004), *Lmx1b*^βgal^/+ (*Lmx1b*-βgal) (Pressman et al., 2000), *Ptf1a*^Cre^/+ (Kawaguchi et al., 2002), *Rosa26*-EYFP (Srinivas et al., 2001), and wild type mice. All mice were bred onto a mixed CD1/C57B6 background. Experiments were done in accordance with the Institute for Laboratory Animal Research Guide for the Care and Use of Laboratory Animals. All experiments were approved by the Animal Studies Committee at Washington University School of Medicine.

### Mapping TF lineages in the medulla

A selected P0 *Atoh1*-Cre; *Rosa26*-stop-yellow fluorescent protein mouse hindbrain (Figure 1F, *Atoh1*-YFP) (Srinivas et al., 2001; Chen et al., 2002) was sectioned at 20 μm into six sets (100 μm separation between sections) from the midbrain to the cervical spinal cord. 4/6 sets were stained for YFP as well as FoxP2, Phox2b, an antibody that recognizes both tyrosine hydroxylase and tryptophan hydroxylase (TH-TPH), or choline acetyltransferase (ChAT). Two-color confocal mosaic images were acquired at 10X for all sections. Reference sections stained for YFP and ChAT were used to manually outline hindbrain sections, motoneuron pools, large *Atoh1*-derived populations, and other major regions visible as background. Sections, co-stained for YFP and FoxP2, Phox2b, or TH-TPH, within 100 μm of each reference section, were aligned, and major populations were manually outlined. Outlines were smoothened for clarity. The purpose was to provide a comparative analysis of the relative location of distinct developmental lineages, not provide a detailed count of cell number.

Digital mosaic images from similarly sectioned P0 *Lmx1b*-βgal and *Dbx1*-βgal hindbrains from approximately identical rostro-caudal regions based on co-expression of ChAT, Phox2b, or FoxP2 were overlain onto reference images and scaled to match size, prior to outlining neuronal populations to define *Lmx1b* and *Phox2b* co-expressing regions as well as *Dbx1*-derived regions. Hindbrain structures were identified by comparison with multiple rodent atlases and existing literature (Kalia and Fuxe, 1985; Kalia et al., 1985a,b,c; Franklin, 1997; Paxinos, 2001; Vanderhorst and Ulfhake, 2006).

### *In situ* hybridization

Slides were immersed in 4% paraformaldehyde (PFA), permeabilized with proteinase K, washed in 0.1 M triethanolamine-HCl with 0.25% acetic anhydride, blocked in hybridization buffer at 65°C, then placed into slide mailers containing hybridization buffer with digoxigenin (DIG) labeled antisense RNA at 1 μg/ml overnight at 65°C. Slides were washed in sodium citrate buffers at 62°C, then washed and incubated in alkaline phosphatase conjugated anti-DIG antibody in 10% normal horse serum and incubated in nitro-blue-tetrazolium chloride and 5-Bromo-4-chloro-3-indolyl phosphate until cellular labeling is clear. For combined IHC and ISH, slides were permeabilized with RIPA buffer and stained for mRNA expression prior to immunohistochemical labeling.

### Genotyping

Mice were genotyped by PCR using primers specific for βgal (5′-GTTGCAGTGCACGGCAGATACACTTGCTGA, 3′-GCCACTGGTGTGGGCCATAATTCAATTCGC), GFP/YFP (5′-GCACGACTTCTTCAAGTCCGCCATGCC, 3′-GCGGATCTTGAAGTTCACCTTGATGCC), Cre recombinase (5′-GCATTACCGGTCGATGCAACGAGTGATGAG, 3′-GAGTGAACGAACCTGGTCGAAATCAGTGCG), *Atoh1* (Ben-Arie et al., 1997), *Dbx1* (Pierani et al., 2001) and *Lmx1b* (Pressman et al., 2000) as described, or by direct visualization of fluorescent reporter or βgal reaction product.

### Tissue acquisition

Neonatal pups (P0–P4) or embryos from timed pregnant females (morning of plug = E0.5) were anesthetized and either perfused (≥E16.5) or immersion fixed in PFA in 0.1 M phosphate buffered saline (PBS), pH 7.4. Embryos or isolated brainstems were postfixed in PFA overnight at 4°C, cryoprotected in 25% sucrose in PBS, blocked, frozen in embedding medium, and stored at −75°C. Perinatal mice were used for several reasons; the vast majority of medullary neurons have reached their adult location making comparison with adult tissue simple, the expression of reporter genes is maximized, and many essential medullary functions, such as breathing, are already active. Brainstems were sectioned in sets of six on a Hacker (Winnsboro, SC) cryostat at 20 μm and sections were thaw mounted onto Superfrost Plus slides and stored at −20°C until use.

### *In situ* hybridization probes

A 717 base pair cDNA fragment of the mouse VGlut2 gene (bps 1268- 2004, NM_080853) was cloned into a PCRII vector (Invitrogen) from mouse cDNA (Cheng et al., 2004). DIG labeled anti-sense *VGlut2* cRNA probes were generated using PCR products as template and T7 RNA polymerase (Roche, Ambion) as previously described (Gray et al., 2010). cRNA probes were purified using Quick Spin columns (Roche) and quantified by spectrophotometry. Probes were used at a concentration of 1 μg/ml. Experiments using sense cRNA probes showed no specific localization.

### IHC and ISH image acquisition

Fluorescent and brightfield images were acquired using a Nikon 90i microscope (Nikon Instruments, Melville, NY), Roper H2 cooled CCD camera (Photometrics, Tucson, AZ), and Optigrid Structured Illumination Confocal with a Prior (Rockland, MA) motorized translation stage. Pseudo-colored images were acquired in Volocity (Perkin Elmer, Waltham, MA), and modified in Photoshop (Adobe, San Jose, CA) or ImageJ (National Institutes of Health, Bethesda, MD) and exported as 8 bit JPEG images. Images were filtered and levels were modified for clarity.

### Antibodies

Chicken anti-beta galactosidase (βgal) 1:4000 (Abcam, Cambridge, MA), Rabbit anti-βgal 1:1000 (Covance, Princeton, NJ), Goat anti-FoxP2 1:2000 (Abcam), Chicken anti-green fluorescent protein (GFP) 1:1000 (Aves Labs, Tilgard, OR), Rabbit anti-GFP 1:2000 (Invitrogen, Carlsbad, CA), Goat anti-Lhx9 1:1000 (Santa Cruz Biotechnology (SCBT), Santa Cruz, CA), Rabbit anti-neurokinin 1 receptor (NK1R) 1:2000 (Advanced Targeting Systems, San Diego, CA, Millipore, Billerica, MA), Rabbit anti-Pax2 1:250 (Invitrogen), Goat anti-Phox2b 1:500 (SCBT), Rabbit anti-Phox2b 1:20,000 (C. Gordis, École Normale Supérieure, Paris, France), Goat anti-somatostatin (SST) 1:600 (SCBT), Rabbit anti-SST 1:500 (SCBT), Guinea pig anti-SST2aR 1:8000 (Gramsch Labs, Schwabhausen, Germany), Sheep anti-tyrosine hydroxylase/tryptophan hydroxylase 1:1000 (Millipore). Chicken anti-TH 1:200 (Aves Labs). Secondary antibodies were species specific and conjugated with FITC, Alexa 488, DyLight 488, Cy3, C5, or DyLight 649 (Invitrogen or Jackson Immunoresearch, West Grove, PA). No specific staining was seen in the absence of any primary antibody.

## Results

Figure 1A is a schematic showing the 16 hypothesized hindbrain progenitor domains divided into eight dorsal (d) and eight ventral (v) domains based on previous work, hypothesized from homologies to spinal cord development, and/or data presented below. The medulla contains 15 of these domains, lacking the dB2 domain (Gaufo et al., 2004; Sieber et al., 2007). Strong evidence for the existence of the dorsal domains has previously been described (Gaufo et al., 2003; Sieber et al., 2007; Gray, 2008; Pagliardini et al., 2008; Rose et al., 2009a; Storm et al., 2009). Within the hindbrain, most ventral domains have not been extensively described and are estimated from published work (Garcia-Campmany et al., 2010; Crone et al., 2012). The neurons of the spinal trigeminal nucleus (SpV) are derived from later born subsets of interneurons similar to what is seen in dorsal spinal cord (Figure 1B) (Glasgow et al., 2005; John et al., 2005; Sieber et al., 2007). Each domain expresses a unique combinatorial pattern of TF expression. For each domain, I have listed a subset of TFs expressed either in progenitor cells (italics) or within post-mitotic neurons (Figure 1A, middle). Additionally, each domain produces neurons with defined neurotransmitter expression although this is the first systematic analysis for many of these populations (Cheng et al., 2004; Glasgow et al., 2005). For example, neurons derived from the dA3 domain are glutamatergic and express the TFs *Lmx1b* and *Phox2b* (Figure 1A, right) (Qian et al., 2001).

In the E12.5 mouse much of hindbrain neurogenesis is complete, however, domains are relatively easy to visualize as both progenitor and newly born postmitotic neurons are present adjacent to the ventricular zone. Figure 1B shows a cartoon of the location of the dA1-v0 progenitor domains and the migration path of subsets of their neural derivatives (color coded as in Figure 1A). Gray box (Figure 1B) and arrows (Figures 1C–E) indicate mixed *Ptf1a*- and *Lmx1b*- derived domains that will form the SpV. Figures 1C–E show the use of combinations of TFs to identify discrete developmental domains. The approximate dorso-ventral lineage boundaries (dA1-v0) are indicated in relation to the location of neurons derived from *Lmx1b* (Figure 1C), *Ptf1a* (Figure 1D), or *Dbx1*-derived lineages (Figure 1E), or expressing FoxP2 (Figures 1C,D) or Phox2b (Figure 1C) proteins. The specific localization of GABAergic neurons is also indicated (Figure 1E). These data point out both the initial strict developmental organization of the hindbrain, but as well as the extensive migration of at least some medullary populations.

The initial laminar development of the hindbrain suggests the majority of interneurons within the hindbrain might remain limited to highly circumspect regions and that neurons derived from different progenitor domains might have little overlap (Figures 1B–E). This has been seen in both xenopus and zebrafish hindbrain (Nikundiwe and Nieuwenhuys, 1983; Ruiz i Altaba and Jessell, 1991; Kinkhabwala et al., 2011; Koyama et al., 2011), but the extent to which this is true in mammals is unclear. To address this, I analyzed the localization of neurons within the perinatal medulla derived from 5 developmental domains: *Atoh1* expressing dA1 (Figure 1F), *Lmx1b* expressing dA3 dB3 and v3l, and *Dbx1* expressing v0 (Figure 1H) interneurons (Figure 1G). Together these data include the locations of neurons from 8 of the 15 medullary progenitor domains: dA1, dA3–dA4, dB3, v0v-c, and v3l and include a large percentage of medullary glutamatergic neurons as well as all aminergic and subsets of cholinergic (see below) and GABAergic neurons (Helms and Johnson, 1998; Pressman et al., 2000; Moran-Rivard et al., 2001; Pierani et al., 2001; Cheng et al., 2004; Gray, 2008; Miesegaes et al., 2009; Rose et al., 2009a). As expected for linage-specific TFs, each gene labeled neurons in discrete locations consistent with previous work. For comparison I also analyzed the localization of a subset of GABAergic neurons in glutamic acid decarboxylase 1-green fluorescent protein (GAD1-GFP) transgenic mice (Figure 1I) (Chattopadhyaya et al., 2004). GABAergic neurons are present throughout most of the medulla.

Figures 1F–I show the localization of gene reporters yellow fluorescent protein (YFP), β-galactosidase (βgal), and GFP for four mouse lines (*Atoh1*-Cre, *Lmx1b*-βgal, *Dbx1*-βgal, and *GAD1*-GFP) in P0 hindbrain at the level of the rostral medulla. In agreement with the highly controlled expression of TFs, the localization of reporter gene expressing cells was consistent in multiple mice indicating that fate-mapping is an efficient way to identify developmentally defined subsets of hindbrain neurons. Importantly, neither *Atoh1* nor *Dbx1* proteins are expressed in post-mitotic neurons. Similarly, *GAD1* protein is largely limited to synaptic terminals. Hence, a fate-mapping approach is the only way to clearly identify these populations. Given their distinct developmental lineages, fate-mapped populations show unique and largely non-overlapping patterns of localization that only partially overlap with *GAD1* expressing regions. For example, *Atoh1*-derived neurons clearly label the external cuneate and linear nuclei (Li, Figure 1F), while *Lmx1b*-derived neurons are present within the NTS and raphe nuclei (Figure 1G). Thus, the localization of significant numbers of medulla neurons is consistent with a limited migration from the progenitor zone. In contrast, all four lineages are also expressed in the ventrolateral medulla and within adjacent intermediate reticular regions. Hence each lineage appears to maintain stereotyped subpopulations with limited dorso-ventral mixing. Some regions, such as the ventrolateral medulla and regions of the reticular formation show possibly overlapping populations. This raises questions as to what extent and where do distinct developmental subpopulations intermingle within the medulla? Answering this might be an important step to define regions of specific lineage for the future understanding of connectivity and gene expression. To address this, I set out to map the regions of expression for each multiple TF lineages onto a single reference brain set.

### Mapping TF lineages in the medulla

**Figure 2:**
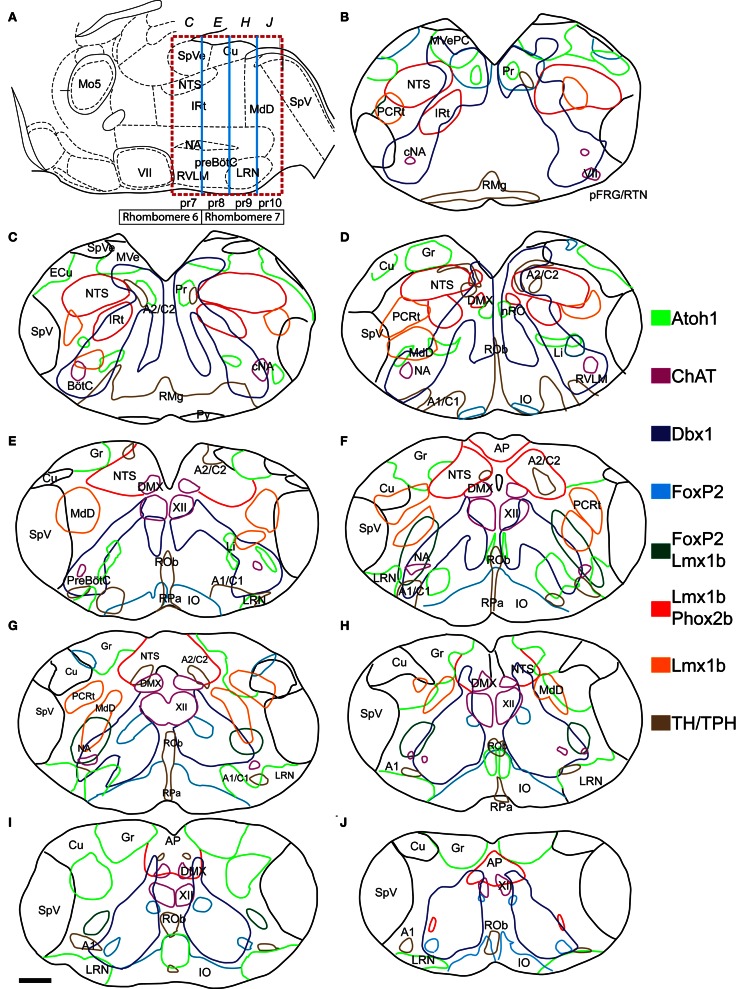
**Atlas of reticular formation developmental origin in neonate mouse medulla. (A)** Mid-sagittal cartoon showing approximate region of mapping (red dashed box). Boxes below hindbrain indicate rhombomere 6 and 7 boundaries. Blue lines indicate pseudo-rhombomere 7–10 boundaries. Italicized letters indicate rostro-caudal position of sections in map. Image modified from Franklin (1997). **(B–J)** Outlines of medullary sections from rostral **(B)** to caudal **(J)** are shown. Sections are separated by 100 μm. Colored lines indicate regions where cells expressing or derived from specific TF lineage are present in P0 mouse (see legend). Red indicates regions of *Lmx1b* and *Phox2b* co-expression. Location of motoneurons and monoaminergic populations are included for reference. Scale bar = 500 μm. See abbreviations for explanation of text.

Figure 2A is a sagittal line drawing of the adult mouse hindbrain modified from Paxino's atlas of the adult mouse brain at the level of the lateral reticular nucleus (LRN) (Franklin, 1997). The approximate boundaries of rhombomeres 6 and 7 are indicated. The caudal boundary of rhombomere 5 is based on the caudal extent of labeling in EGR2 (early growth factor 2, aka Krox20) transgenic mouse lines (Voiculescu et al., 2001; Manzanares et al., 2002). The rostral boundary of rhombomere 7 is based on the rostral extent of HoxA4 expression while the caudal boundary is an estimate based on size (Rivkin and Cordes, 2008; Huang et al., 2012). Figures 2B–J outlines the regions where distinct developmentally defined cells are located within the neonatal medulla at approximately 100 μm rostro-caudal resolution. Identified regions are indicated (see abbreviations). *Atoh1*-, *Dbx1*-, and *Lmx1b*- derived populations are present along the entire length of the medulla. For example, the dA3 *Lmx1b*/*Phox2b* population that specifies NTS and medullary catecholaminergic neurons has no direct homolog in the cervical spinal cord and ends near the hindbrain/spinal boundary (Figure 2J). *Atoh1*- and *Dbx1*- derived populations both continue to the sacral spinal cord (not shown). In general, the localization of developmentally defined populations are clearly not organized within a strictly nuclear organization as within the thalamus, nor are the populations largely intermingled as is seen in spinal cord (Helms and Johnson, 2003; Gray et al., 2004; Garcia-Campmany et al., 2010).

**Figure 3:**
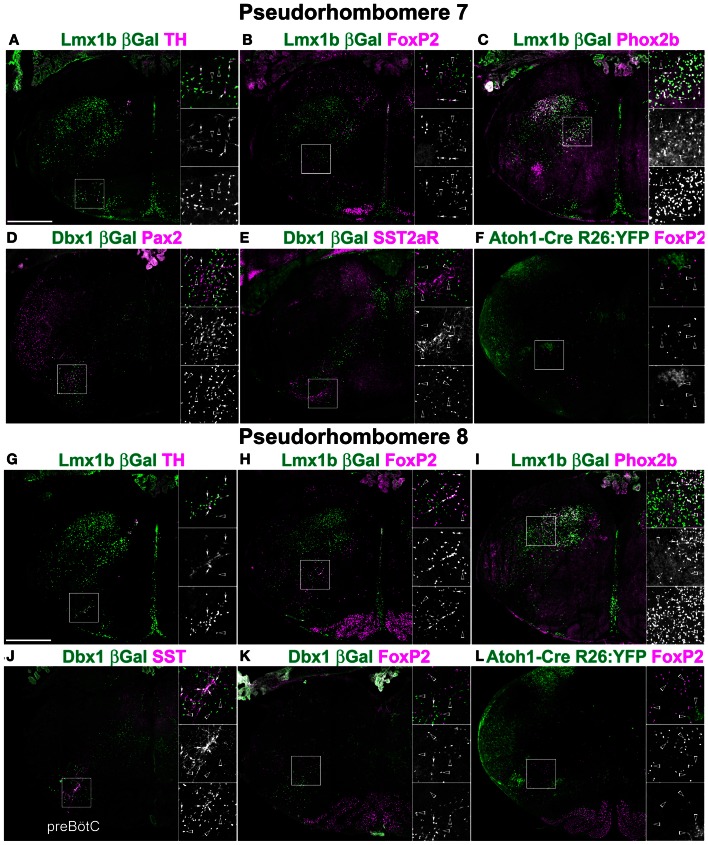
**Segmental organization of combinatorial patterns of gene expression within rostral medulla hindbrain lineages.** Pseudo-color fluorescent confocal mosaic hemi-sections from P0 transgenic mice at the level of pseudo-rhombomere 7 **(A–F)** and 8 **(G–L)**. Images from *Lmx1b*-βgal **(A–C,G–I)**, *Dbx1*-βgal **(D,E,J,K)**, and *Atoh1*-Cre; R26YFP **(F,L)** mice showing localization of lineage reporter (green) β-Gal **(A–E,G–K)** or YFP **(F,L)** with reference genes (magenta) TH **(A,G)** FoxP2 **(B,F,H,K,L)** Phox2b **(C,I)** Pax2 **(D)**, SST2aR **(E)**, or SST **(J)**. Boxed region in each image is expanded to right showing overlapping (top), and single colors magenta (middle), and green (bottom). Arrows indicate co-expression. Arrowheads indicate absence of co-expression. See Figures 2C,E for identification of specific regions. Scale bar = 500 μm.

The medulla contains many populations identifiable using Nissl staining (Altman and Bayer, 1980a,b; Kalia and Fuxe, 1985; Vanderhorst and Ulfhake, 2006). Most of these also correspond to populations where a single lineage is predominant. Several of these populations are located adjacent to their progenitor domains, (Figures 1B,C) consistent with limited migration, and show limited population mixing. For example, *Atoh1*-derived, dA1 neurons of the vestibular, gracilis, and cuneate nuclei lie dorsal to the subset of dA3 *Lmx1b*- and *Phox2b*-derived neurons that form the NTS (Figures 2C–H). Note that by P0, not all *Lmx1b*-derived NTS neurons continue to express Phox2b likely due to down regulation of *Phox2b* protein as is seen in other populations (Figure 3C) (Pattyn et al., 1997; Dubreuil et al., 2000). Other populations with known patterns of migration such as the dA1, *Atoh1*-derived pre-cerebellar glutamatergic neurons of the LRN (Figures 2E–J) and their continuation the Li (Figures 2C,D) (Rose et al., 2009a), as well as the dA4, FoxP2 expressing IO are also clearly defined (Yamada et al., 2007; Storm et al., 2009). In addition, portions of the prepositus hypoglossi and nucleus of Roller (Pr, nRO, Figures 2B,D) are also dA1, *Atoh1*-derived populations as previously described (Fu et al., 2009; Rose et al., 2009a; Ray et al., 2011). The nRO and Pr lie near the midline and projects to cerebellum similar to the *Atoh1*-derived, pre-cerebellar neurons in the spinal cord (Brodal, 1952; Miesegaes et al., 2009).

### Defining the reticular formation

**Figure 4:**
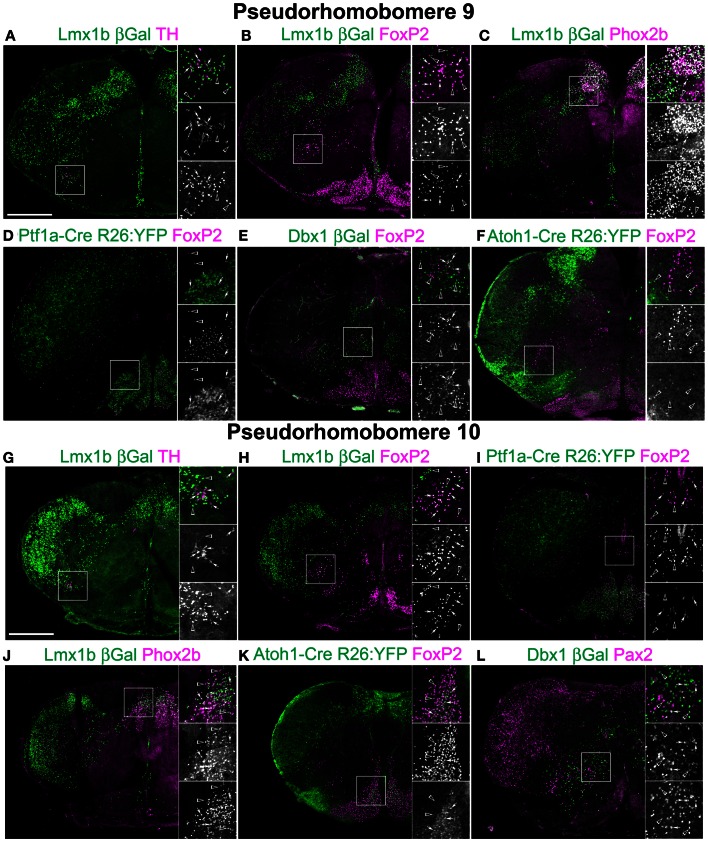
**Segmental organization of combinatorial patterns of gene expression within caudal medullary hindbrain lineages.** Pseudo-color fluorescent confocal mosaic hemi-sections from P0 transgenic mice at the level of pseudo-rhombomere 9 **(A–F)** and 10 **(G–L)**. Images from *Lmx1b*-βgal **(A–C,G,H,J)**, *Ptf1a*-Cre; R26YFP (D, I) *Dbx1*-βgal **(E,L)**, and *Atoh1*-Cre; R26YFP **(F,K)** mice showing localization of lineage reporter (green) βGal **(A–C,E,G,H,J,L)**, or YFP **(D,F,I,K)** with reference genes (magenta) TH **(A,G)**, FoxP2 **(B,D–F,H,I,K)**, Phox2b **(C,J)**, or Pax2 **(L)**. Arrows indicate co-expression. Boxed regions are expanded to right showing overlapping (top), and single colors magenta (middle), and green (bottom). Scale bar = 500 μm.

Outside of the clearly identifiable medullary regions, i.e., within the reticular formation, the localization of developmentally defined populations remained highly stereotyped. In addition to the NTS, *Lmx1b*, and *Phox2b* are co-expressed in both dorsal and ventral TH expressing neurons (Figures 2, 3A,G, 4A,G, 6C,G), as previously described (Pattyn et al., 1997, 2000a; Qian et al., 2002). *Lmx1b* and *Phox2b* also define the non-TH expressing intermediate reticular formation (IRt) at rostral medullary levels (Figures 2C,I). These neurons have been shown to express *Phox2b* in the adult rat, but their co-expression with *Lmx1b* suggests they are developmentally related to the NTS and may be involved in secondary viscerosensory processing (Kang et al., 2007).

The reticular zone just medial to the spinal nucleus of the trigeminal nerve (SpV) has been shown to be important for the coordination of pain and eye blink reflex pathways (Almeida and Lima, 1997; Almeida et al., 1999; Lima and Almeida, 2002; Leite-Almeida et al., 2006; Smit et al., 2006), and receives direct cortical and spinal input (Desbois et al., 1999). *Lmx1b*-derived neurons were present within the parvocellular reticular formation and dorsal medullary reticular nucleus (PCRt, MdD, Figures 2B–H). Neither of these populations co-expressed Phox2b (Figures 3I, 4C). This suggests these populations may be derived from later born *Lmx1b*-derived neurons that migrate to the SpV and may correspond developmentally and possibly functionally to late-born, deep dorsal horn neurons in the spinal cord (Ding et al., 2004; Glasgow et al., 2005).

The distribution of v0, *Dbx1*-derived neurons is less localized to discrete regions, but still shows a clear spatial organization. A subpopulation within the rostral medulla is located lateral to their developmental origin (Figures 2B,C). It lies ventral to the vestibular nuclei and medial to the NTS and largely overlapping to the Pr (Figures 3E,F) with scattered neurons extending ventrally. This suggests *Dbx1*-derived neurons make up a significant part of the Pr nuclei although whether these are glutamatergic or GABAergic is unknown (McCrea et al., 1979). In the spinal cord, the majority of *Dbx1*-derived neurons migrate ventro-medially toward the midline (Pierani et al., 2001; Lanuza et al., 2004). A similar population is present within the mid to caudal medulla extending into the spinal cord (Figures 2C–J). In the spinal cord, these largely GABAergic neurons have been proposed to play a role in coordinating alternating activity during locomotion (Lanuza et al., 2004).

*Dbx1*-derived neurons are also present within a narrow stripe extending from the dorsomedial to ventrolateral surfaces of the medulla. Within the medial medulla, this population is clearly separate from the medially migrating population described above (Figures 2C–F, 3D,E,J,K). Within the intermediate reticular formation (IRt), *Dbx1*-derived neurons form a narrow stripe that partially overlaps with *Lmx1b*/*Phox2b* neurons (Figures 2B–D) but does not overlap with the dorsal medullary reticular nucleus (MdD) neurons (Figures 2E–H). Within the caudal medulla (Figures 2G–J, 4E,L), the stripe of *Dbx1*-derived neurons is less apparent due to both the size of the population and the more compact area of the medulla.

The ventrolateral medulla contains neurons from multiple progenitor domains including catecholaminergic dA3, *Lmx1b*/*Phox2b*- derived neurons, small populations of undescribed FoxP2 neurons, and dA1, *Atoh1*-derived neurons. It also contains an expansion, relative to the spinal cord, of the v0v, *Dbx1*-derived stripe forming the neurons of the respiratory column (Gray et al., 2010). This developmental variety is consistent with previous results regarding the heterogeneity of ventral respiratory and cardiovascular control regions and will be discussed in more detail below.

Figure 2 also includes the localization of *FoxP2. FoxP2* is co-expressed by several different lineages. However, this protein and its expression are highly conserved in rats and mice and persist in adult rat brain (Alvarez et al., 2005; Stein and Loewy, 2010; Geerling et al., 2011; Miller et al., 2011, 2012; Shin et al., 2011). *FoxP2* was previously found expressed in subsets of *Atoh1*- and *Lmx1b*-derived pontine neurons as well as several subpopulations of spinal cord neurons (Alvarez et al., 2005; Prasad et al., 2008; Geerling et al., 2011; Miller et al., 2012). The availability of high-quality, commercially available antibodies makes it a good marker for defining specific hindbrain populations. FoxP2 is expressed within the *Ptf1a*-derived, dA4 population that forms the IO in nearly every neuron (Figures 2D–J, 3H, 4E,I). It is also expressed within a subset of vestibular nuclei neurons (Figure 3B) and throughout the medulla in small, scattered populations (Figures 3H, 4B,H). As FoxP2 was expressed along the entire length of the medulla, its co-localization was analyzed in *Atoh1*-, *Dbx1*-, and *Lmx1b*-derived populations. FoxP2 was not co-expressed with Phox2b within the medulla. FoxP2 was also not expressed in medullary dA1, *Atoh1*-derived neurons (Figures 3F,L, 4F,K) except for a small number of the caudal most IO neurons. This latter co-expression may represent leakage of Atoh1-driven recombinase expression into the most caudally derived IO neurons (Chen and Keens, 2004; Tsuda et al., 2005). FoxP2 was co-expressed in a small subset of *Lmx1b*-derived neurons in the ventral medulla (Figures 3B,H, 4B,H) from the rostral medulla to the spinal cord border with the population becoming more apparent at caudal levels. Based on their location relative to other *Lmx1b* populations, these neurons likely represent a subset of dB3 interneurons. FoxP2 was not expressed in *Dbx1*-derived neurons within the ventral medulla, but was co-expressed in a subset of neurons ventrolateral to the XII motor nucleus (Figure 4E) similar to spinal cord (Prasad et al., 2008).

### Combinatorial coding and the pseudo-rhombomeric model

These data are consistent with the hypothesis that the organization of the medullary reticular formation is a direct consequence of developmental lineage. Previous work has shown much of pontine organization is the direct result of early segmental-like patterning (Krumlauf et al., 1993). Puelles and colleagues, working in chick, proposed the medulla is similarly patterned and consists of multiple pseudo-rhomobomeres with specific neuronal populations being derived from specific rostro-caudal regions of the early neuroepithilum (Cambronero and Puelles, 2000; Marin et al., 2008). Based on homology with chicken hindbrain, Figure 2A includes the approximate pseudo-rhombomeric boundaries. Whether this organization is present within mouse hindbrain is unclear.

As yet, no TFs have been identified in the mouse with rostral or caudal boundaries that correspond to the proposed pseudo-rhombomeric boundaries similar to Hox genes. In the chick the LRN and IO are proposed to be derived from pseudo-rhombomere 9 (pr9) progenitors. In the mouse, both Li and the LRN are *Atoh1*-derived and have been proposed to be continuous with Li neurons sitting dorsal to the nucleus ambiguous (NA) and LRN on the ventral surface (Fu et al., 2009). Li neurons, however, are limited to the approximate region of pr7 along with the compact formation of the NA (cNA) with a diffuse transition until it forms the LRN in pr9 (Figures 3F,L, 4F,K). Thus, at some basic level, there is extensive anatomical conservation within the medulla between species at least within highly recognizable structures. If this hypothesis is accurate, however, it should be possible to find distinct differences in gene expression between developmentally related neurons over short rostro-caudal distances.

**Figure 5:**
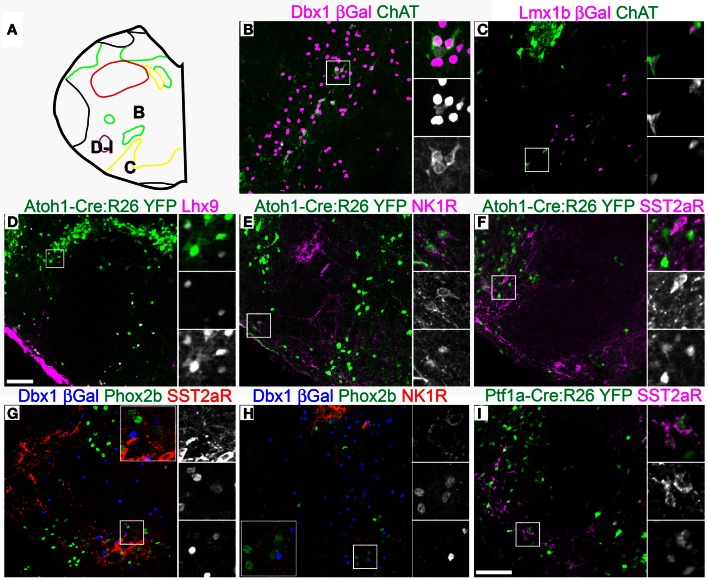
**Pseudo-rhombomere 7 specific patterns of gene expression.** Pseudo-color fluorescent confocal mosaic images from P0 transgenic mouse at the level of pseudo-rhombomere 7. **(A)** Schematic image modified from Figure 2C indicating location of co-expressing populations shown in **(B–I)**. (**B–I**) Images from *Dbxl*-βgal **(B,G,H)**, *Lmx1b*-βgal **(C)**, *Atoh1*-Cre; R26YFP **(D–F)** and *Ptf1a*-Cre; R26YFP **(I)** mice showing localization of lineage reporter β-gal (**B,C**, magenta, **G,H**, blue), or YFP (**D–F**, green) with ChAT (**B,C**, green), Lhx9 (**D**, magenta), NK1R (**E**, magenta, **H**, red), SST2aR (**F,I**, magenta, **G**, red), and *Phox2b* (**G,H**, green). Boxed regions in **(B–F,I)** are expanded to right showing overlapping (right, top), and single colors magenta (middle), and green (bottom). Boxed regions in **(G,H)** are expanded (inset) with single colors red (right, top), green (middle), and blue (bottom). Scale bar = 500 μm.

**Figure 6:**
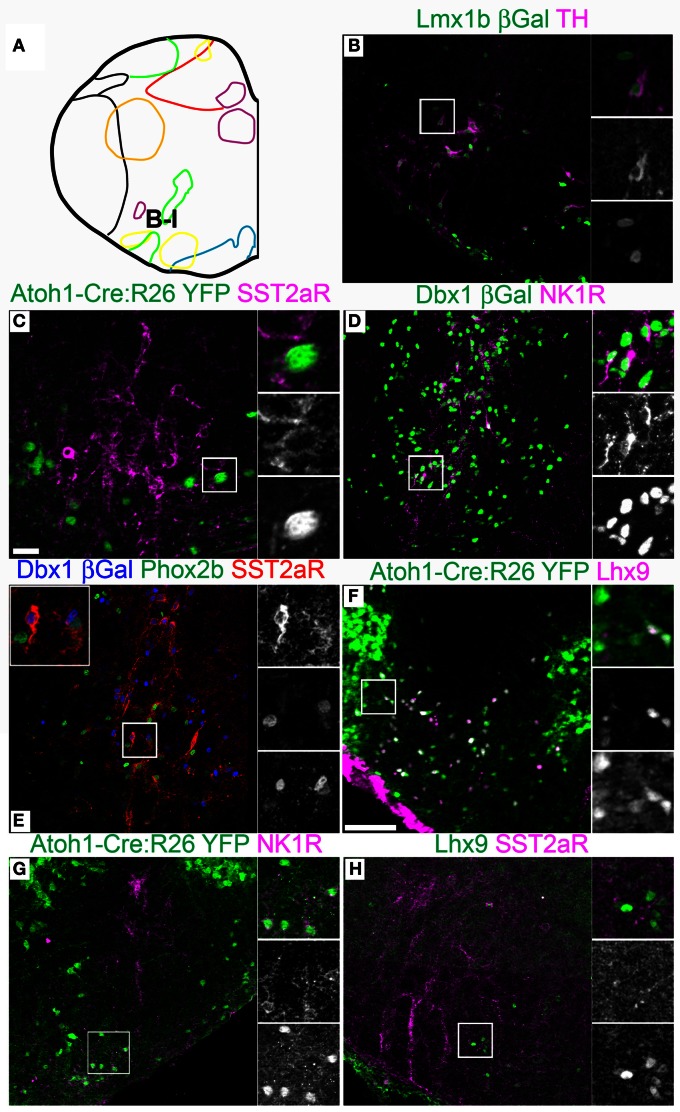
**Pseudo-rhombomere 8 specific patterns of gene expression.** Pseudo-color fluorescent confocal mosaic images from P0 transgenic mouse at the level of pseudo-rhombomere 8. **(A)** Schematic image modified from Figure 2E indicating location of co-expressing populations shown in **(B–H)**. **(B–H)** Images from *Lmx1b*-βgal **(B)**, *Atoh1*-Cre; Rosa26YFP **(C,F,G)**, *Dbx1*-βgal **(D,E)**,and wild type **(H)** mice showing localization of lineage reporter (green) βgal (**B,D**, green, **E**, blue), or YFP **(C,F,G)** with TH **(B)**, SST2aR **(C,E,H)**, NK1R (**E,H)**, Phox2b **(E)**, and Lhx9 **(F,H)**. Text color indicates gene color in images. Boxed region in **(B–D,F–H)** is expanded to right showing overlapping (right, top), and single colors magenta (middle), and green (bottom). Boxed region in **(E)** is expanded (top) with single colors red (right, top), green (middle), and blue (bottom). Scale bar = 500 μm.

**Figure 7:**
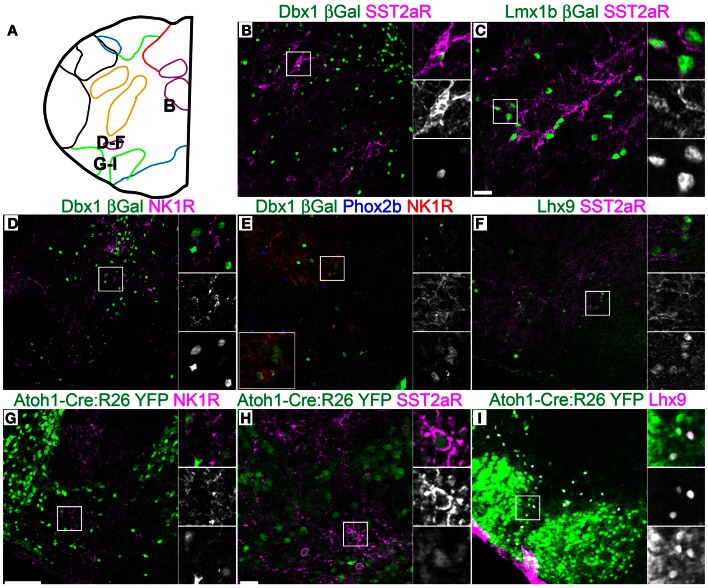
**Pseudo-rhombomere 9 specific patterns of gene expression.** Pseudo-color fluorescent confocal mosaic images from P0 transgenic mouse at the level of pseudo-rhombomere 9. **(A)** Schematic image modified from Figure 2G indicating location of co-expressing populations shown in **(B–I)**. (**B–I)** Images from *Dbx1*-βgal **(B,D,E)**, *Lmx1b*-βgal **(C)**, wild type **(F)**, and *Atoh1*-Cre; R26YFP **(G–I)** mice showing localization of lineage reporter (green) β-gal **(B–E)** or YFP **(G–I)** with SST2aR **(B,C,H)**, NK1R **(D,E,G)**, or Lhx9 **(I)**. Text color indicates gene color in images. Boxed region in **(B–E,G–I)** is expanded to right showing overlapping (right, top), and single colors magenta (middle), and green (bottom). Boxed region in **(E)** is expanded (bottom left) with single colors red (right, top), green (middle), and blue (bottom). Scale bar = 500 μm.

To address whether pseudo-rhombomeric organization might provide a useful way to analyze the organization of the medulla, the combinatorial expression of TF lineage was analyzed to determine whether discrete subpopulations might be genetically identifiable. Figures 3, 4 show confocal mosaic hemisections of *Lmx1b*, *Ptf1a*, *Dbx1*, and *Atoh1* transgenic reporter mice co-stained with various antibodies for comparison. Sections correspond to approximate pseudo-rhombomeric boundaries (pr7; Figures 3A–F, pr8; Figures 3G–L, pr9; Figures 4A–F, pr10; Figures 4G–L). In contrast to this hypothesis, several populations were found whose locations were either continuous across the hindbrain or were more consistent with existing rhombomeric populations. *Atoh1*-derived neurons consist of at least two distinct subpopulations. First are pre-cerebellar neurons such as the LRN that express the TF BarH-like 1 (BarHL1) and express vesicular glutamate transporter 1 (VGlut1) (Bermingham et al., 2001; Gray et al., 2004). Second are small populations of interneurons that express the related TFs *Lhx9* and *Lhx2* (lim homeobox 2, 9) and vesicular glutamate transporter 2 (VGlut2). In the ventral medulla, the *VGlut2* and *Lhx9* expressing subset of interneurons are ventral to *Dbx1*- and *Lmx1b*-derived populations and are in addition to the *Atoh1*-derived Li and LRN neurons (Figure 6C) (Helms and Johnson, 2003; Gray et al., 2010; Machold et al., 2011). In the pr7 region, these dA1 neurons are scattered surrounding the rostroventrolateral medulla (RVLM) region (Figure 5D). In pr8–9 they are located ventral to *Dbx1*-derived and *Lmx1b*-derived glutamatergic neurons, overlying the LRN (Figures 6C,F-G, 7I) (Gray et al., 2010). Similarly, a subset of Dbx1-derived cells expresses Pax2 across the entire ventral medulla (Figures 3D, 4L). In neither case is there any obvious segmental organization. The *Lmx1b*/*Phox2b* neurons of the IRt extend to the approximate rostral rhomobomere 7 boundary (Figures 2B–D, 3C), and *Lmx1b*/*FoxP2* expressing neurons extend the approximate length of rhombomere 7 (Figures 2F–H, 4B,H).

Alternately, it is possible that segmental organization may not appear as the generation of novel hindbrain populations, but is present as differences in gene expression within developmentally related populations. Because of the importance of populations within the ventrolateral medulla in homeostatic control, the expression patterns of multiple genes have been analyzed in some detail providing an additional test. I analyzed the expression of ChAT, somatostatin peptide (SST), SST2a receptor (SST2aR), and neurokinin 1 receptor (NK1R) across the medulla (Pilowsky, 2008; Pilowsky et al., 2009). The hindbrain contains a number of distinct populations of cholinergic interneurons, most notably within the rostral pons (Vanderhorst and Ulfhake, 2006). At least two distinct subpopulations of ChAT expressing interneurons are present within the rostral medulla exclusively within the pr7 region (Figure 5A). One population is *Dbx1*-derived and is dorsal to the cNA (Figure 5B). A second population is *Lmx1b*-derived and lies within the pr7 region (Figure 5C). This region overlaps the RVLM region important for control of vasomotor tone (Chalmers et al., 1992, 1994; Guyenet, 2006; Stornetta et al., 2012). This population does not express *FoxP2* or *Phox2b*.

Strong cell-body IHC expression of SST peptide is limited to a subset of *Dbx1*-derived, preBötC interneurons (Figure 3J) (Stornetta et al., 2003; Llona et al., 2004; Llona and Eugenin, 2005; Bouvier et al., 2010; Gray et al., 2010). While the preBötC abuts the approximate rostral boundary of rhombomere 7 (Figures 2A,E, 6A), its caudal boundary is more consistent with the pr8/pr9 than rhombomere 7/8 boundary (Figure 7A). SST2aR is expressed on distinct subsets of neurons throughout the ventral medulla as well as other regions (Schindler et al., 1997; Burke et al., 2008; Gray et al., 2010). *Lmx1b*-derived TH neurons co-express SST2aR across the ventral medulla with no clear boundary (Figure 7C) (Burke et al., 2011). Within *Dbx1*-derived neurons, SST2aR expression is more complex. It is not expressed on *Dbx1* neurons in pr7, but is expressed on *Dbx1* neurons within the preBötC (pr8, Figure 6E) as well as within the rVRG (pr9, Figure 7B). Within the preBötC, SST2aR is co-expressed with SST peptide indicating a possible auto-regulatory role (Gray et al., 2010).

Lhx9 expressing neurons dorsal to the LRN (pr9) express SST2aR (Figures 7F,H) but more rostral (pr7–8) neurons do not (Figures 5F, 6H). Within pr7, r *Dbx1*-derived V0 neurons do not express SST2aR (Figure 5G). *Lmx1b*- and *Phox2b*-derived, TH expressing dA3 neurons, however, do express SST2aR, but they represent only a subset of SST2aR neurons (Figure 5G). Recently, a population of non-catecholaminergic, glutamatergic neurons that express high levels of SST2aR within the RVLM of the rat was identified (Burke et al., 2008). Based on anatomy and pharmacology, it was proposed these neurons are fundamental for the generation of the basal vasomotor tone, but their relationship to other brainstem SST2aR expressing populations was unknown. An identical population is present in the mouse RVLM (Figure 5I). This population of SST2aR expressing neurons is derived from *Ptf1a* expressing progenitors and is completely limited to the RVLM/ventral pr7 region (Figures 5F,G,I). This is surprising as *Ptf1a*-derived neurons are almost exclusively inhibitory within the medulla except for *FoxP2* expressing glutamatergic, IO within pr9–10 (Figures 4D,I). These data suggest that the functionally identified region represent a previously unidentified developmental population consistent with a specific functional role.

This pattern of limiting peptide receptor expression to subsets of neurons within specific rostro-caudal population is not ubiquitous. NK1R is expressed on *Dbx1*-derived neurons throughout the ventral medulla, although its expression is highest within the preBötC (Figures 5H, 6D, 7D,E) (Gray et al., 1999, 2010; Guyenet et al., 2002). Similarly, NK1R is expressed on a subset of *Lhx9* expressing, *Atoh1*-derived neurons across multiple pseudo-rhombomeres (Figures 5E, 7G) (Rose et al., 2009b; Gray et al., 2010). Thus, while NK1R expression is not regionally restricted, it is clearly co-localized with other genes in a region specific manner.

### Transmitter identity

One major component of neural identity, neurotransmitter, is a direct consequence of developmental program. The expression of neurotransmitter generating enzymes such as glutamic acid decarboxylases (*GAD1-2*), or transmitter specific transporters such as the vesicular glutamate transporters (*VGlut1-3, Slc17A6-8*) begins co-incident with the specification of neural identity (Cheng et al., 2004). In the hindbrain, all three VGlut proteins are present, however, *VGlut1* is largely limited to the subset of *Atoh1*-derived pre-cerebellar subpopulations such as the LRN, and *VGlut3* is expressed in a subset of caudal raphe neurons (Hisano et al., 2002; Stornetta et al., 2002a,b; Boulland et al., 2004; Fremeau et al., 2004; Stornetta et al., 2005). Hence, *VGlut2* (*Slc17A6*) is the major enzyme coordinating a glutamatergic phenotype in the hindbrain. The data presented suggest neurons derived from different developmental lineages migrate into specific anatomical regions. This raised the question whether TF lineage could identify distinct glutamatergic populations.

**Figure 8:**
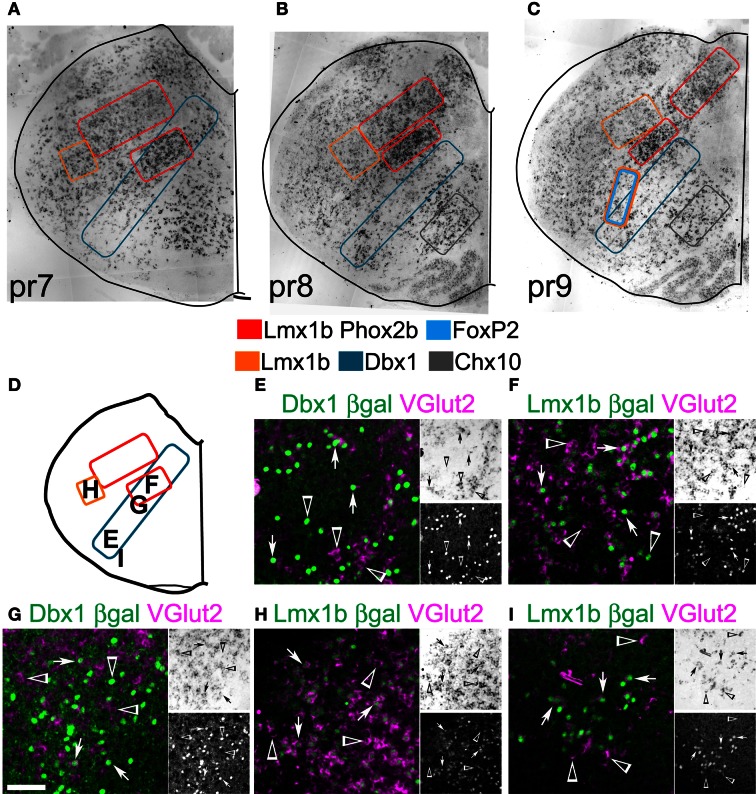
**Developmental origin of medullary reticular formation glutamatergic neurons.** Brightfield *in situ* hybridization of *VGlut2* mRNA from P0 mouse brain overlain with modified outlines from brain outlines from Figures 2C (**A**, pr7), **2E** (**B**, pr8), and **2G** (**C**, pr9). Colored boxes indicate populations derived from distinct developmental lineages (see legend). **(D)** Schematic image modified from Figure 2C indicating location of glutamatergic populations of pr7 shown in **(E–I)**. **(E–I)** Images showing inverted VGlut2 mRNA (magenta) and βgal (green) from *Dbx1*-βgal **(E,G)**, and *Lmx1b*-βgal **(F,H,I)**. Single color images shown to right. VGlut2 (top, brightfield), β-Gal (bottom). Arrows indicate co-expression. Arrowheads indicate absence of co-expression. Scale bar = 500 μm.

**Figure 9:**
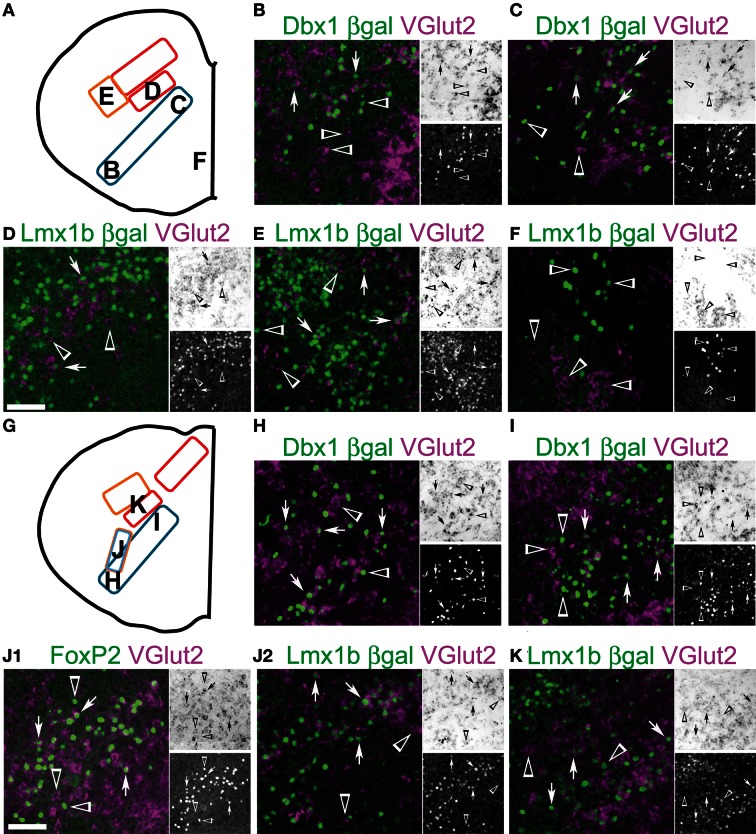
**Developmental origin of medullary reticular formation glutamatergic neurons.** Schematic image modified from Figure 2E **(A)** or from Figure 2G **(G)** indicating location of glutamatergic populations of pr8 **(B-F)** or pr9 **(H-K)**. **(B-F, G-K)** Images showing inverted VGlut2 mRNA (magenta), βgal (green), and FoxP2 **(J1**, green**)** from Dbx1-βgal **(B, C, H, I)**, Lmx1b-βgal **(D-F, J2-K)** and wild type **(J1)** mice. Note **(J1)** and **(J2)** are from the same region from different animals. Single color images shown to right. VGlut2 (top, brightfield), βGal (bottom). Arrows indicate co-expression. Arrowheads indicate absence of co-expression. Scale bar = 500 μm.

Figures 8A–C shows (ISH) for *VGlut2* at three levels of the medulla corresponding to pseudo-rhombomeres 7–9 (Figures 8D, 9A,G). Schematic outlines indicate the relative positions of neurons derived from *Dbx1*, *FoxP2*, *Lmx1b*, and/or *Phox2b* lineages. *Chx10* (ceh-10 homeodomain containing homolog 10) is a defining TF for the v2a population and has recently been shown to be essential for a large medial *VGlut2* expressing population (estimated in Figures 8B,C) (Crone et al., 2012). As indicated in Figure 1, these TFs are expressed in subsets of glutamatergic neurons, but also in other populations including GABAergic, catecholaminergic, cholinergic, and serotonergic neurons. To more directly test whether glutamatergic populations correspond to specific developmental lineages, combined ISH for *VGlut2* and IHC for reporter genes was performed in the medulla.

Within pr7, the majority of NTS glutamatergic neurons are *Lmx1b*- and *Phox2b*- derived (not shown). The lateral MdD, Lmx1-derived population shows a high percentage of *Lmx1b* derived glutamatergic neurons (Figure 8H). Some of the remaining glutamatergic neurons may be dA1, *Atoh1*-derived neurons (Figure 1F) (Rose et al., 2009a). Ventral to the NTS, the IRt region has a high density of glutamatergic neurons derived from both *Dbx1* and *Lmx1b* lineages (Figures 8F,G). Within this region, most *Lmx1b*-derived are glutamatergic. *Dbx1*-derived neurons consist of a mixture of glutamatergic, GABAergic, and cholinergic neurons as well as glia.

The RVLM contains both *Dbx1*- and *Lmx1b*-derived glutamatergic neurons (Figures 8E,I). Most dA3, *Lmx1b*-, and *Phox2b*-derived neurons co-express VGlut2 and TH, as in rat (Stornetta et al., 2002b). Nearly 50% of *Dbx1*-derived cells co-express VGlut2. The majority of non-glutamatergic *Dbx1*-derived βgal expressing cells, within this region, are glia (Fogarty et al., 2005; Gray et al., 2010).

Within pr8 (Figure 9A), the *Lmx1b*-derived glutamatergic population lateral to the NTS persists (Figure 9E). Ventral to the NTS, the IRt region contains a continuation of the pr7 dA3, *Lmx1b*-, and *Phox2b*-derived population as well as a smaller unlabeled additional population (Figure 9D). These unknown neurons are not *Dbx1*-derived and they do not express FoxP2. Based on their location, they may be a subset of *Ptf1a*-derived, dA4 interneurons, although this remains to be shown.

At this level, *Dbx1*-derived glutamatergic neurons extend from directly lateral to the XIIn to the ventral medullar surface (Figures 9C,H) and show limited overlap with other populations (Figures 9B,C). The dorsal region corresponds with regions of XII pre-motor neurons identified by tracing and calcium imaging studies (Dobbins and Feldman, 1995; Koizumi et al., 2008). The ventrolateral population corresponds to glutamatergic preBötC respiratory neurons. Serotonergic *Lmx1b*-derived neurons do not express *VGlut2* as previously shown (Figure 9F) (Ding et al., 2003).

Figure 9G shows the schematic from Figure 8C and indicates the location of pr9 populations. Similar to pr7 and pr8, *Dbx1* glutamatergic neurons are present ventrolateral to dorsomedial (Figures 9H,I) and both the NTS and dorsal population of *Lmx1b* derived neurons are still present (Figure 9K). Pr9–10 contains a large population of *FoxP2* expressing neurons in the ventral reticular formation (Figures 2F–H, 4B,H). These neurons are nearly all glutamatergic (Figure 9J1) and most are *Lmx1b* derived (Figure 9J2). The *Lmx1b*/FoxP2 expressing neurons likely represent previously undescribed population of dB3 interneurons.

## Discussion

The medulla is a complex structure consisting of multiple heterogeneous populations in close proximity. Outside of populations recognizable by either Nissl stain or by the small number for which genetic markers have been identified, the overall organization of this vital brain region has remained largely unknown. In contrast, during early development, the different classes of neurons are formed in an orderly fashion along the neural tube. I hypothesized that aspects of this early developmental organization persist and form the basis for an orderly pattern of neural organization in the developed medulla.

Focusing on developmental progenitor populations that generate glutamatergic neurons, I used a fate-mapping strategy in multiple transgenic mouse lines coupled with ISH and IHC to identify and map populations of neurons derived from *Atoh1*, *Dbx1*, and *Lmx1b* expressing lineages. Briefly, the major finding is that the medulla is structurally organized in both the dorso-ventral and rostro-caudal axes. Except for some specific exceptions (see below), glutamatergic neurons of the medullary reticular formation are organized into a series of dorso-ventral stripes that correspond to paths of migration from early progenitor domains. These stripes only partially overlap. Within a single developmentally derived stripe, however, there are clear differences in gene expression between ventral and dorsal regions.

Rostro-caudally, similar differences in gene expression within a developmental domain roughly correlate with hypothesized pseudo-rhombomes identified in avian brainstem (Cambronero and Puelles, 2000; Marin et al., 2008). The presence of specific developmentally derived populations, such as the IRt and v0c cholinergic neurons (see below), provides some evidence consistent with this model. Also consistent are the boundaries of peptide receptor expression within *Dbx1* neurons of the ventrolateral medulla. The difficulty is that in the absence of clear genetic boundary markers similar to that shown in pons such as *Egr2* (*Krox20*) (Voiculescu et al., 2001; Manzanares et al., 2002), these descriptions remain somewhat anecdotal in mammals. Regardless, however, the pseudo-rhombomeric model allows for a reproducible and understandable way of defining the medulla that should allow for a better estimation of anatomical location for both this work and future studies.

Within the approximate pr7 region (Figures 2B,C) there are multiple previously unknown or under described populations. Just ventral to the NTS, the IRt has previously been described as a *Phox2b* expressing population in adult rat (Kang et al., 2007). Based on fate-mapping, I show that this glutamatergic population (Figures 8F, 9D) is derived from the dA3, *Lmx1b*, and *Phox2b* expressing domain similar to the NTS, although it does not receive direct vagal input from the periphery.

Ventral to IRt and dorsomedial to the cNA is a population of cholinergic interneurons derived from *Dbx1* expressing progenitors (Figure 5B) that may be similar to *Pitx2* expressing, Dbx1-derived, v0c interneurons that make large C-boutons onto spinal motoneurons. In spinal cord, these neurons are important for the cholinergic modulation of motoneuron function and they make large c-bouton synapses onto lumbar motoneurons (Miles et al., 2007; Zagoraiou et al., 2009; Miles and Sillar, 2011). Unlike the spinal cord, however, these neurons are not located adjacent to the 4th ventricle, the medullary equivalent of the central canal. Previous work suggests *Pitx2* is not expressed within the hindbrain (Diez-Roux et al., 2011), but these neurons may be the source of large cholinergic boutons present on cranial motoneurons in pig and mouse brainstem including the XII. These neurons are present in adult rat where a subset has been shown to project to XIIn (Travers et al., 2005).

Ventral to the cNA, in addition to the well-known RVLM catecholaminergic and BötC glycinergic neurons there are several additional populations. First, a small population of cholinergic neurons are present adjacent to lateral portion of the raphe magnus and just caudal to the VIIn (Figure 5C). The functional role of this population is unknown although it has recently been shown to project to brain regions important for sensory, but not autonomic, respiratory, or endocrine regulation (Stornetta et al., 2012). Additionally they may be responsible for fast cholinergic EPSPs found in cNA motoneurons (Zhang et al., 1983) or cholinergic modulation of NTS or respiratory function (Bieger, 2001; Shao and Feldman, 2009). Given their location adjacent to the raphe, this population may be derived from the V3l, *Lmx1b* domain that also produces serotoninergic neurons (Cheng et al., 2003; Ding et al., 2003; Craven et al., 2004; Pattyn et al., 2004; Alenina et al., 2006; Zhao et al., 2006).

The RVLM has long been known to contain glutamatergic populations modulating vasomotor tone, the most well-known being *Lmx1b*-derived catecholaminergic neurons (Figure 6B). It was hypothesized some subset of these neurons might be responsible for generating basal tone as ablation of this region blocks neurogenic hypertension (Varner et al., 1994; Pilowsky et al., 2009), although the ablation of spinally projecting catecholaminergic has only limited effect on basal blood pressure (Guyenet et al., 2001; Madden and Sved, 2003). Burke and colleagues identified, in adult rat, a small population of non-catecholaminergic neurons, spinally projecting, SST2aR expressing, glutamatergic neurons and showed that local application of SST into the RVLM caused dose-dependent sympathoinhibition and hypotension (Burke et al., 2008). They proposed these neurons play a role in generating vasomotor tone. Interestingly, the mouse RVLM also contains a possibly equivalent SST2aR expressing population (Figures 5F,G,I). These neurons are a small subpopulation of *Ptf1a* derived interneurons (possibly dA4, Figure 5I). Nearly all *Ptf1a*-derived neurons, however, are inhibitory with only the IO known to express *VGlut2* (Figures 4D,I) (Yamada et al., 2007). As the IO is derived from more caudal brainstem structures, I propose these SST2aR neurons represent a novel population of glutamatergic *Ptf1a* derived neurons limited to the RVLM consistent with previous hypotheses of a distinct, non-catecholaminergic, glutamatergic population in control of blood pressure (Lipski et al., 1995; Schreihofer and Guyenet, 1997).

The proposed pr8 region (Figures 2D,E) contains a continuation of the *Lmx1b* and *Phox2b* derived IRt interneurons (Figures 8B, 9D). Ventrolateral to the NTS are glutamatergic neurons of the PCRt and MdD (Figures 8A–C,F). Many of these neurons are *Lmx1b*, but not *Phox2b* derived suggesting they may represent a subset of late born neurons derived from the progenitor domain from which the SpV forms (Figures 1B,C) and be similar in development and function to deep dorsal horn glutamatergic neurons (Helms and Johnson, 2003). These neurons extend to the medullary/spinal boundary consistent with this possible developmental origin. Neurons within the region containing dorsal *Lmx1b*, PCRt population have been implicated in facilitating nociceptive behavior (Almeida et al., 1999). These neurons receive ascending innervation from dorsal horn projection neurons as well as descending input from multiple cortical areas (Almeida and Lima, 1997; Desbois et al., 1999). Previously, neurons within the MdD region been implicated in the eye blink reflex as they express cFos after supraorbital nerve stimulation and project to the orbicularis oculi muscle innervating VII motoneurons (Smit et al., 2006).

Ventral to the scNA are the subset of glutamatergic SST, SST2aR, and NK1R expressing respiratory neurons of the preBötC (Figures 3J, 6D,E). These neurons are a subset of the column of v0v, *Dbx1*-derived glutamatergic neurons essential for breathing. Recent work suggests this population overlaps the rostral-most region of rhombomere 7, although the exact relationship between preBötC SST neurons and rhombomeric and pseudo-rhombomeric boundaries is still unclear. Most ventrally is the most rostral border of the dA4, *Ptf1a* derived IO neurons.

One additional population that is most apparent at the pr8 level is a dorsal continuation of glutamatergic v0v, *Dbx1*-derived neurons that extends toward the dorsal midline (Figures 8B, 9C). At this level, there is a complete segregation between the *Dbx1* and *Lmx1b* populations. This population, however, extends from pr7 to at least pr9. Rostrally, it overlaps with Lmx1b-derived IRt population (Figures 8A,F,G).

The proposed pr9 region (Figures 2F–H) contains several distinct populations. Most dorsal is the *Lmx1b* and *Phox2b* expressing Area Postrema (AP, Figures 2F, 4J). The AP includes several distinct subpopulations including a population of *hydroxysteroid dehydrogenase 2* and *Phox2b* expressing, glutamatergic neurons activated by salt-deprivation (Geerling and Loewy, 2008). Ventral to the NTS is a continuation of the *Lmx1b* derived PCRt and MdD populations that begin in pr8. At this level is the first appearance of a newly described population of *Lmx1b*-derived, FoxP2 expressing neurons that extends into pr10 (Figures 2F–I, 4B,H). This population of glutamatergic neurons, likely because of its small size and relative sparse distribution, has no currently known function. Based on its location, I hypothesize these neurons are dB3, *Lbx1*-, and *Lmx1b*-derived.

The location of the *Lmx1b*/*Phox2b* derived dA3, IRt population overlaps with regions previously identified as important for viscerosensory integration. In adult rats and mice, quinine induces strong cFos immunoreactivity in both the NTS and within the ventral IRt population in both GABAergic and non-GABAergic populations (Dinardo and Travers, 1997; Travers et al., 2007). This region receives direct cortical input and is reciprocally connected to gustatory centers within the NTS and parabrachial nuclei (Norgren, 1978; Herbert et al., 1990; Shammah-Lagnado et al., 1992; Dinardo and Travers, 1997; Travers et al., 1997). In total, there are four major dA3, *Lmx1b*/*Phox2b* derived populations; the AP, NTS, IRt, and catecholaminergic A1–2/C1–2. Historically, the NTS has been regarded primarily as the way station for transmitting visceral sensory input to higher processing centers. These findings suggest the dA3 population may be capable of much more extensive and independent function than largely assumed.

Within the intermediate reticular formation, the majority of glutamatergic neurons between the NTS dorsally and ventral medullary surface are derived from only two lineages, the *Lmx1b*- and *Phox2b*-derived dA3 and the *Dbx1*-derived v0v. In the ventrolateral medulla these populations overlap with the v0v, *Dbx1* neurons being essential for respiratory output and the catecholaminergic and glutamatergic dA3, A1/C1 neurons being important for coordinating responses to stress. Dorsally, these populations overlap in the IRt at the pr7 level, but are clearly segregated at more caudal levels.

Ventral to the dA3, IRt populations lies the stripe of *Dbx1*-derived neurons, many of which are glutamatergic, v0v neurons (Figures 8A,E). With the pr7 region, this population overlaps with glutamatergic premotor neurons that project to both XII and V motor pools (Dinardo and Travers, 1997). Neurons within this region are strongly rhythmically active during licking but not swallowing and expressed cFos with oral quinine administration (Travers et al., 1997). Within pr8–9, dorsal v0v glutamatergic populations are located in the region known to contain presumptive glutamatergic premotor neurons of the XIIn based on calcium imaging and trans-synaptic labeling experiments (Dobbins and Feldman, 1995; Fay and Norgren, 1997; Koizumi et al., 2008). As these neurons are developmentally related to neurons essential for respiratory rhythm generation, it suggests the *Dbx1* population may play an essential role in rhythmic motor output for multiple behaviors, not just breathing (Bouvier et al., 2010; Gray et al., 2010).

The pattern of organization I describe here is, at least in part, consistent with proposals put forward by Johnson over 100 years ago (Johnson, 1902) and which have been recently described in the organization of transmitter populations in zebrafish (Kinkhabwala et al., 2011; Koyama et al., 2011). It is also partially consistent with Nieuwenhuys' topographical description of hindbrain organization (Nieuwenhuys, 2011) with the addition of a more direct testing of the relationship between developmental and adult location. It is important to note, however, that this proposed schematic has several limitations. Dorso-ventrally it is most consistent with populations within the intermediate reticular formation. The ventrolateral medulla, in contrast, shows extensive mixing of multiple developmental populations (dA1, dA3, dB1, dB3, and v0v) while the dorsal medulla contains multiple subpopulations within clearly defined nuclear structures, such as the NTS with some variation at the boundaries depending upon location.

Combined with the discrete progenitor domains present during early development, I hypothesize the medulla is organized into a “reticular lattice” with clear dorso-ventral and rostro-caudal regions of shared development, gene expression, and possibly function. The developmental origins of diverse reticular formation populations can be defined by anatomical location and neurotransmitter phenotype. Broadly, the fate-mapping data described here combined with previous connectivity and activity data from both rats and mice suggest both a genetic and anatomical separation between sensory and motor processing. While Johnson proposed this general hypothesis over a century ago (Johnson, 1902), here I show that the two major glutamatergic lineages, dA3 and v0v, are adjacent to each other, in spite of their distant developmental origins. Whether this linkage between viserosensory and motor outputs is evolutionarily conserved is unknown.

### Conflict of interest statement

The author declares that the research was conducted in the absence of any commercial or financial relationships that could be construed as a potential conflict of interest.

## Abbreviations

A1/C1: A1/C1 catecholaminergic group
A2/C2: A2/C2 catecholaminergic group
Ach: acetylcholine
AP: area postrema
Ascl1: achaete-scute homolog 1
Atoh1: atonal homolog 1
βgal: beta-galactosidase
bHLHb5: basic helix loop helix b5
BötC: Bötzinger Complex
ChAT: choline acetyltransferase
Chx10: ceh-10 homeodomain containing homolog
cNA: compact Nucleus Ambiguus
Cre: cre-recombinase
Cu: cuneate nucleus
Dbx1: developing brain homeobox 1
DMX: dorsal motor nucleus of the vagus
ECu: external cuneate
En1: engrailed 1
Evx1: evenskipped 1
FoxP2: forkhead box P2
GABA: gamma aminobutyric acid
GAD1: glutamic decarboxylase 1
GATA3: GATA binding protein 3
Glu: glutamate
GFP: green fluorescent protein
Gly: glycine
Gr: gracile nucleus
IO: inferior olive
IRt: internal reticular formation
Lbx1: ladybird homeobox 1
Lhx9: LIM homeobox 9
Li: linear nucleus
Lmx1b: LIM homeobox transcription factor 1 beta
LRN: lateral reticular nucleus
MdD: dorsal reticular nucleus of the medulla
Mo5: trigeminal motor nucleus
MVe: medial vestibular nucleus
MVePC: medial vestibular nucleus parvocellular
Ngn1: neurogenin 1
NKx2.2: NK2 transcription factor related, locus 2
nRO: nucleus of Roller
NA: nucleus ambiguus
NK1R: neurokinin 1 receptor
NTS: nucleus of the tractus solitarius
Olig1: oligodendrocyte transcription factor 1
Pax2: paired box 2
PCRt: parvocellular reticular region
Pet1: plasmacytoma expressed transcript 1
pFRG: parafacial respiratory group
Phox2b: paired-like homeobox 2b
Pitx2: paired-like homeodomain transcription factor 2
Pr: prepositus hypoglossi
Pr7–10: pseudo-rhombomere 7–10
preBötC: preBötzinger Complex
Ptf1a: pancreas specific transcription factor, 1a
Py: pyramidal tract
RMg: raphe magnus
RPa: raphe palidus
ROb: raphe obscurus
RTN: retrotrapezoid nucleus
R26: Rosa26
RVLM: rostroventrolateral medulla
SpV: spinal nucleus of the trigeminal nerve
SpVe: spinal vestibular nucleus
SST: somatostatin
SST2aR: somatostatin 2a receptor
TF: transcription factor
TH: tyrosine hydroxylase
TPH: tryptophan hydroxylase
VII: facial motonucleus
VGlut2: vesicular glutamate transporter 2 (SLC17A6)
XII: hypoglossal motonucleus
YFP: yellow fluorescent protein
5-HT: serotonin.
